# The relations among foreign language anxiety, academic buoyancy and willingness to communicate in EFL classroom

**DOI:** 10.3389/fpsyg.2025.1634054

**Published:** 2025-10-28

**Authors:** Yuxin Lin, Baochang Wang, Guizhen Zhang, Yankui Su

**Affiliations:** College of Foreign Languages, Fujian Normal University, Fuzhou, China

**Keywords:** academic buoyancy, foreign language anxiety, willingness to communicate in EFL classroom, structural equation modeling, mixed-methods

## Abstract

This study, grounded in the frameworks of Positive Psychological Resources Theory and Emotion Regulation Theory, investigated the mechanism through which foreign language anxiety (FLA) and academic buoyancy (AB) influence students' willingness to communicate (WTC) in EFL classroom. A mixed-methods approach was employed, combining a Structural Equation Modeling (SEM) analysis based on data from 627 senior high school students and semi-structured interviews with eight randomly selected participants in China. The empirical results indicate that FLA significantly and negatively predicts WTC, while AB plays a partial mediating role in this relationship, thereby validating the proposed “FLA—AB—WTC” path model. These results highlight academic buoyancy as a positive psychological trait that mediates the effect of anxiety on willingness to communicate. This research offers theoretical and practical implications for affective regulation and psychological empowerment in foreign language pedagogy.

## 1 Introduction

Willingness to communicate (WTC) has long been regarded as a core construct in second language acquisition (SLA), with substantial implications for learners' communicative success and overall language achievement (Clément et al., 2003). Since the proposal of the pyramid model (MacIntyre et al., 1998), which conceptualizes WTC as the outcome of multilayered influences ranging from social context to individual affective and personality factors, increasing attention has been given to how variables such as anxiety, enjoyment, growth mindset, and L2 grit shape learners' readiness to engage in second language communication (Bensalem et al., 2025a; Wang, 2023; Xu and Zhang, 2025). Among these, foreign language anxiety (FLA) has consistently emerged as a key factor that hinders learners' oral expression and willingness to participate in classroom interaction (Bielak, 2025; Horwitz et al., 1986; Tsang and Dewaele, 2024; Zheng et al., 2025).

Although existing research has consistently shown that FLA negatively predicts WTC (Kalsoom et al., 2020), much of the current literature remains limited to single-path analysis, lacking a more integrative approach that incorporates variables across different levels of the pyramid model. In particular, little attention has been paid to how this prototypical negative emotion interacts with personality-related traits in shaping communicative behavior. With the growing influence of positive psychology in SLA research, academic buoyancy (AB), defined as a learner's capacity to effectively cope with everyday academic challenges, has been introduced as a promising individual trait that may help explain this relationship from a strengths-based perspective.

Unlike traditional deficit-oriented views, positive psychology emphasizes the activation of internal psychological resources to help individuals manage emotional difficulties and sustain engagement (Ryff, 2022). As a key personal strength, academic buoyancy has been shown to encompass emotion regulation capacities (Martin and Marsh, 2008). Notably, the “composure” dimension of its 5Cs framework (Martin et al., 2010) is often interpreted as an indicator of low anxiety, which theoretically supports its potential to mediate the link between anxiety and communication-related outcomes by fostering emotional stability.

Despite its theoretical relevance, the potential mediating role of AB in the relationship between anxiety and WTC remains underexplored in SLA. In particular, few studies have examined whether academic buoyancy can mitigate the communication barriers induced by language anxiety in classroom settings. To address this gap, the present study draws on both Positive Psychological Capital Theory (Luthans et al., 2007) and Emotion Regulation Theory (Gross, 1998), arguing that positive personal traits such as AB may reshape the adverse impact of negative emotions by fostering emotional resilience and sustaining learners' willingness to communicate.

By positioning academic buoyancy as a mediating variable, this study proposes a path model “FLA—AB—WTC” to explore how positive psychological traits serve as psychological conduits that transmit emotional influences and support adaptive functioning in second language learning contexts. This model not only reflects the theoretical convergence between positive psychology and emotion regulation perspectives but also contributes to a more nuanced understanding of how emotional and dispositional factors jointly shape communicative behavior. Furthermore, it offers practical insights for designing affective interventions in EFL classrooms aimed at fostering emotionally resilient and communicatively engaged learners. As highlighted in Wong's existential positive psychology (2022), the essence of positive development lies not in avoiding anxiety, but in transforming it into strength through struggle and meaning-making, a view that resonates with the path explored in this study and informs its pedagogical implications. In doing so, this study contributes a novel trait-based perspective grounded in positive psychology to the growing body of research on affective variables and classroom WTC.

## 2 Literature review

### 2.1 Willingness to communicate in EFL classroom

(McCroskey and Baer 1985) originally defined willingness to communicate (WTC) as an individual's probability of choosing to initiate communication, conceptualizing it as a relatively stable personality trait in first language (L1) contexts. This perspective, however, shifted when (MacIntyre et al. 1998) extended the concept to second language (L2) learning, defining WTC as a state of readiness to engage in conversation with specific interlocutors using the L2 at a particular moment. Their definition highlighted the situational and dynamic nature of WTC in L2 contexts, which contrasts with its trait-like conceptualization in L1 settings. To explain this complexity, they proposed the “pyramid model” of L2 WTC, which outlines six interrelated layers, ranging from social and contextual factors to personality traits, affective states, and motivational orientations, providing a comprehensive framework for subsequent empirical inquiry (MacIntyre et al., 1998).

Building on this foundation, recent studies have begun to explore a range of psychological factors that influence willingness to communicate (WTC), particularly emotional variables (Kalsoom et al., 2020) and personality-related traits (Ebn-Abbasi et al., 2024). Within the framework of positive psychology, empirical research can be broadly categorized into several strands. Some studies have focused solely on dispositional variables such as L2 grit, growth mindset, and self-efficacy (Bensalem et al., 2025a,b; Chen et al., 2025; Hejazi et al., 2023; Ho et al., 2025; Xu and Zhang, 2025). Others have examined emotional and dispositional factors in parallel, investigating their respective contributions to WTC (Bensalem et al., 2025a; Fu, 2025). A third line of work has considered anxiety and enjoyment, two contrasting emotional variables, as mediators linking emotional states with personality traits (Li, 2025; Sadoughi and Hejazi, 2024; Zarrinabadi et al., 2024). In addition, some research has centered on the relationship between positive traits and positive emotions (Yang et al., 2024). Despite these efforts, the interplay between negative emotions and positive dispositional traits in shaping WTC remains underexplored and lacks systematic empirical attention. To address this issue, the present study focuses on two representative constructs, foreign language anxiety (an emotional factor) and academic buoyancy (a personality trait), to investigate their potential interplay and collective impact on students' classroom WTC.

Moreover, WTC is not a fixed disposition but rather a context-sensitive construct that fluctuates depending on situational factors (Kang, 2005; Lee and Liu, 2024). For most high school students, the classroom represents the primary context in which they engage with a foreign language. Therefore, this study specifically situates WTC within the classroom environment to enhance the practical relevance and contextual applicability of its findings.

### 2.2 Foreign language anxiety

(Horwitz et al. 1986) were among the first to conceptualize foreign language anxiety (FLA) as a distinct category of anxiety, separate from general academic anxiety. FLA refers to a complex emotional reaction, including feelings of tension, worry, and avoidance, that arises in classroom-based language learning contexts due to learners' self-perceptions, beliefs, and the communicative demands of the environment (Horwitz et al., 1986). As a cognitively and emotionally draining negative factor, FLA has been consistently shown to interfere with second language learning processes (Horwitz, 2001). In recent years, research on FLA has expanded significantly, covering topics such as individual difference factors, multidimensional emotional experiences, learning performance, and learning outcomes (Zhang and Han, 2024).

In second language learning, positive and negative emotions often coexist and interact in complex ways (Oxford, 2016a,b). Drawing on this insight, positive psychology emphasizes the activation of internal psychological resources to help individuals better manage challenges and regulate emotions, and asserts that positive psychological constructs may play a key role in mediating the influence of negative emotional states by supporting emotional regulation and recovery (Abbas et al., 2024). However, within SLA research, studies that simultaneously incorporate both positive and negative psychological variables into a single theoretical model remain relatively limited (Fathi et al., 2021). Most existing work has focused on the contrast between enjoyment and anxiety, often modeling them in parallel paths (Alanazi and Bensalem, 2024; Derakhshan et al., 2025; Li, 2025), while largely neglecting how positive personality traits might mediate the influence of negative emotional responses through indirect emotional regulation mechanisms.

Although prior studies have confirmed that foreign language anxiety negatively predicts learners' willingness to communicate (Kalsoom et al., 2020), the underlying mediating mechanisms remain insufficiently explored, particularly in terms of how positive psychological resources might mediate the relationship by transforming negative affect into constructive behavioral outcomes.

### 2.3 Academic buoyancy

Academic buoyancy (AB), introduced by (Martin and Marsh 2008) within the framework of positive psychology, refers to students' ability to effectively cope with everyday academic challenges and setbacks. It emphasizes the development of learners' adaptability and psychological resilience in ordinary educational settings by drawing on their positive psychological resources. While academic resilience primarily concerns how a small subset of students responds to major adversities, academic buoyancy is more relevant to the broader population of students who experience frequent, low-intensity stressors as part of their daily school life (Martin and Marsh, 2008). As such, AB reflects a more ecologically valid construct that captures the psychological demands of most learners. It also aligns closely with positive psychology's focus on fostering adaptive traits in typical individuals, rather than solely targeting those in crisis (Seligman and Csikszentmihalyi, 2000). In this sense, AB may offer greater research and practical value than resilience, particularly in mainstream educational contexts.

AB reflects students' capacity for self-regulation and psychological adjustment in response to routine academic stress, and is considered instrumental in helping them remain emotionally stable and cognitively engaged amid the fluctuations and uncertainties of the learning process (Datu and Yuen, 2018). In recent years, academic buoyancy has attracted growing scholarly attention across general education settings, with research exploring both internal traits and external supports that influence its development (Collie et al., 2016; Derakhshan and Fathi, 2025; Granziera et al., 2024; Rafsanjani et al., 2024; Zhi et al., 2024). However, due to its domain-specific nature (Martin and Marsh, 2008), the functional mechanisms of AB may vary across academic disciplines.

Compared with other subject areas, language learning relies more heavily on communicative practice and intercultural competence. This process entails not only knowledge acquisition but also emotion regulation, identity negotiation, and sustained willingness to express oneself in a second language (Dewaele and MacIntyre, 2016). In this context, the emotional stability and adaptive strategies associated with academic buoyancy may serve as crucial psychological resources that enable learners to overcome foreign language anxiety and communication-related barriers.

Although a number of recent studies, both domestic and international, have begun to examine AB in the context of second language acquisition (Fathi et al., 2025; Fu, 2024; Hedeshi and Ghanizadeh, 2025; Liu, 2022; Souzandehfar and Ahmed Abdel-Al Ibrahim, 2023), empirical research in this area remains limited. Given that students with higher levels of AB are more capable of maintaining composure in the face of everyday academic difficulties, this trait may not only contribute to emotional regulation in the face of foreign language anxiety but also foster a more stable psychological state and proactive coping tendency. These qualities, in turn, may positively influence learners' classroom WTC. Nevertheless, the potential mediating role of academic buoyancy in the relationship between FLA and WTC has yet to be systematically investigated.

### 2.4 Relationships among foreign language anxiety, academic buoyancy, and willingness to communicate in the EFL classroom

In recent years, numerous empirical studies have consistently confirmed the negative predictive effect of foreign language anxiety on learners' willingness to communicate, making this relationship one of the more stable findings in the field of second language acquisition (Kalsoom et al., 2020; Liu, 2018). However, within the ongoing shift toward positive psychology in SLA research, scholars have tended to model anxiety and positive emotions as opposing constructs in parallel pathways (Alanazi and Bensalem, 2024; Derakhshan et al., 2025; Li, 2025), with relatively few efforts made to investigate how anxiety, as a representative negative emotion, might be processed and reshaped through internal psychological mechanisms. While growing attention has been devoted to the facilitative role of positive emotions in language learning (Bensalem et al., 2025a; Fu, 2025), far less is known about how positive psychological traits may mediate the relationship between anxiety and learning outcomes by supporting adaptive emotional processing. Few studies have attempted to systematically model this interaction.

Against this backdrop, academic buoyancy, as a representative positive psychological trait, was originally conceptualized with the implicit assumption that anxiety could negatively impact learners' ability to remain buoyant (Martin et al., 2010). Recent studies have begun to provide preliminary empirical support for a possible path from FLA to AB in second language contexts (Liu et al., 2025). However, such studies remain limited in scope, and the potential role of AB as a regulatory resource in the language learning process remains underexplored. The present study thus seeks to introduce academic buoyancy as a mediating variable, with the goal of expanding existing anxiety-related models beyond purely emotional perspectives and offering a more trait-based explanatory framework.

As a psychological resource that helps individuals cope with routine academic stress, academic buoyancy supports learners' adaptive functioning and emotional regulation, which may in turn positively influence learning-related behaviors (Yu et al., 2019). While the direct relationship between academic buoyancy and classroom willingness to communicate has not been extensively discussed, early evidence from (Khajavy et al. 2019) demonstrated that psychological capital, a construct comprising hope, optimism, self-efficacy, and resilience, significantly predicted L2 WTC among Iranian university students, with resilience showing a positive correlation with WTC (r = 0.23, *p* < 0.01). A later study by (Trigueros et al. 2024) found that academic resilience further supported the role of resilience by showing that it positively predicted willingness to communicate among Spanish secondary school students. Most recently, (Abdullaeva et al. 2024) provided experimental evidence that academic resilience significantly predicted students' willingness to communicate in AI-assisted EFL assessment environments. Their findings also indicated that resilience can be fostered through technology-enhanced interventions, thereby promoting both emotional stability and communicative engagement under assessment pressure. These findings collectively point to the relevance of resilience in enhancing WTC, thereby strengthening the rationale for examining related constructs like academic buoyancy. Although buoyancy and resilience are conceptually related, the former is more relevant to the daily academic challenges faced by most students and, within the framework of positive psychology, may offer greater theoretical coherence and practical relevance.

(Luthans et al. 2007), integrating psychological capital theory with the broaden-and-build framework, proposed a four-dimensional structure of psychological capital, comprising hope, optimism, self-efficacy, and resilience, which underscores the role of internal resources in promoting sustained cognitive and behavioral engagement. Academic buoyancy, reflecting students' stable coping capacity and psychological flexibility in the face of everyday academic challenges (Martin and Marsh, 2008), may be understood as a manifestation of these broader positive psychological resources. Academic buoyancy enables learners to maintain emotional balance under anxiety-inducing conditions, thereby mitigating the disruptive effects of negative emotions on learning-related behaviors.

Beyond psychological capital, Seligman's (2011a,b) PERMA model of wellbeing further underscores the relevance of academic buoyancy in the context of positive psychology. The PERMA framework highlights five pillars, Positive Emotion, Engagement, Relationships, Meaning, and Accomplishment, as the building blocks of wellbeing. Academic buoyancy can be aligned with these dimensions: by helping learners sustain positive emotions under stress, remain engaged in academic tasks, foster supportive peer relationships, pursue meaning in classroom experiences, and celebrate incremental accomplishments. In this sense, buoyancy not only reflects resilience against everyday academic challenges but also contributes to broader student wellbeing. Moreover, (Seligman 2011a,b) emphasized resilience as central to flourishing, a view that resonates with buoyancy's adaptive role in transforming academic setbacks into opportunities for psychological growth and communicative engagement.

Further, emotion regulation theory (Gross, 1998) posits that individuals can employ various cognitive and behavioral strategies to manage their emotional states, reduce the interference of negative emotions, and sustain psychological equilibrium. In the 5C model of academic buoyancy proposed by (Martin et al. 2010), the dimension of Composure captures the emotional regulation aspect of the construct. It refers to students' ability to stay calm and psychologically steady in anxiety-inducing contexts such as exams, which essentially involves managing emotional responses. Thus, from the inception of the construct, emotional regulation has arguably been embedded as a core, though implicit, component of academic buoyancy. Empirical studies have further supported the close relationship between the two (Alazemi et al., 2023; Heydarnejad et al., 2022; Kritikou and Giovazolias, 2022). In this light, academic buoyancy functions as an adaptive and restorative response to academic stress and may be interpreted as a form of emotional regulation in itself. Learners with high levels of buoyancy often possess stronger emotional self-regulation capacities, allowing them to manage anxiety more effectively and thus reduce its detrimental impact on classroom willingness to communicate. This, in turn, provides a psychological foundation for more confident expression and interaction in foreign language classrooms.

In addition, the Affective Filter Hypothesis (Krashen, 1985) suggests that affective states function as a filter in language acquisition, wherein negative emotions such as anxiety may hinder the reception and processing of language input and interfere with communicative performance. From this perspective, the emotional stability and regulatory strength associated with academic buoyancy may help lower the affective filter, facilitate more efficient language input processing, and ultimately enhance learners' willingness to communicate in classroom settings.

### 2.5 Hypothetical model

In summary, while previous research suggests potential associations among foreign language anxiety (FLA), academic buoyancy (AB), and willingness to communicate (WTC) in the EFL classroom, the specific relationships and directional pathways among these variables have yet to be clearly articulated. Drawing on the aforementioned theoretical frameworks and empirical findings, the present study proposes a conceptual model in which academic buoyancy functions as a mediating variable between foreign language anxiety and classroom WTC.

Specifically, it is hypothesized that foreign language anxiety negatively affects students' willingness to communicate, whereas academic buoyancy positively predicts WTC. Moreover, academic buoyancy is expected to attenuate the negative impact of anxiety on WTC by serving as an intermediary psychological resource. The hypotheses are as follows:

H1: Foreign language anxiety negatively predicts willingness to communicate in the EFL classroom.
H2: Academic buoyancy mediates the relationship between foreign language anxiety and willingness to communicate.

## 3 Methodology

### 3.1 Participants

With approval from a top-tier senior high school in Fujian Province, China, this study recruited 683 Grade 11 students as participants. The secondary school stage is a critical period of physiological and psychological development, during which students undergo marked transitions in learning capacity (Ma et al., 2015). Compared to junior high school students, senior high school students typically possess a more systematic knowledge of English and demonstrate more mature language abilities. Grade 11 students, in particular, have completed 1 year of high school English instruction, making their second language proficiency relatively stable. Furthermore, as they have not yet entered the high-pressure final year of university entrance exam preparation, they represent a group with both high assessability and generalizability.

The data were collected using anonymous questionnaires administered on site, with a 20-min time limit. A total of 627 valid responses were obtained, resulting in an effective response rate of 91.8%. Among the respondents, 330 were male (52.6%) and 297 were female (47.4%). In terms of academic track, 523 students (83.4%) were from the science stream, and 104 (16.6%) from the humanities stream. The average age of the participants ranged from 16 to 17 years, and all had received at least 9 years of English education with no overseas learning experience.

### 3.2 Research instruments

As part of the mixed-methods design, this section outlines the instruments used for both quantitative and qualitative data collection. Specifically, it introduces the paper-based questionnaires and the semi-structured interview protocol employed in the study. All instruments were administered in their Chinese-translated versions. Each item was rated using a five-point Likert scale, ranging from 1 (“strongly disagree”) to 5 (“strongly agree”).

#### 3.2.1 Foreign language anxiety scale

Foreign language anxiety was measured using the Foreign Language Classroom Anxiety Scale (FLCAS) originally developed by (Horwitz et al. 1986) and translated into Chinese (Wang, 2003). The scale has demonstrated good psychometric properties. Confirmatory factor analysis showed an acceptable model fit: χ^2^/df = 2.637 (within the acceptable range of 1–3), CFI = 0.950, TLI = 0.946, RMSEA = 0.051 (< 0.08), and SRMR = 0.0321 (< 0.08), indicating satisfactory structural validity.

The scale includes four dimensions: communication apprehension, fear of negative evaluation, test anxiety, and general classroom anxiety. The Cronbach's alpha coefficients for these four subscales were 0.957, 0.950, 0.849, and 0.915, respectively. The overall reliability of the scale was high, with a total Cronbach's α of 0.965.

#### 3.2.2 Academic buoyancy scale

Academic buoyancy was assessed using the unidimensional scale developed by (Martin and Marsh 2008) and revised by (Sun 2009) for use in the Chinese educational context. The scale exhibited satisfactory structural validity based on the following model fit indices: χ^2^/df = 2.084 (within the acceptable range of 1–3), CFI = 0.998, TLI = 0.994, RMSEA = 0.042 (< 0.08), and SRMR = 0.0099 (< 0.08). The scale also demonstrated good internal consistency, with a Cronbach's α of 0.867.

#### 3.2.3 Willingness to communicate scale

Students' willingness to communicate in the EFL classroom was measured using the instrument developed by (Wang 2019). Confirmatory factor analysis showed an acceptable model fit: χ^2^/df = 3.061 (approaching the acceptable threshold), CFI = 0.963, TLI = 0.957, RMSEA = 0.057 (< 0.08), and SRMR = 0.0331 (< 0.08), indicating sound structural validity. The scale consists of four sub-dimensions: teacher–class communication (α = 0.907), teacher–individual communication (α = 0.929), peer communication (α = 0.889), and group communication (α = 0.912). The total scale demonstrated excellent internal consistency, with a Cronbach's α of 0.946.

In addition to the subscale coefficients reported above, we further examined the measurement properties by computing composite reliability (CR), McDonald's ω, and average variance extracted (AVE). As summarized in Table 1, all α and ω values exceeded the 0.80 benchmark, CR values were above the recommended threshold, and AVE values were greater than the conventional 0.50 cut-off. Collectively, these indices provide strong evidence of internal consistency and satisfactory convergent validity across all constructs.

**Table 1:** Reliability and Convergent Validity (*N* = 627).

**Construct**	**Items**	**AVE**	**CR**	**McDonald's ω**	**Cronbach's α**
Communication apprehension (CA)	11	0.669	0.957	0.957	0.957
Fear of negative evaluation (FNE)	11	0.635	0.950	0.951	0.950
Test anxiety (TA)	3	0.655	0.851	0.850	0.849
General classroom anxiety (GCA)	8	0.574	0.915	0.915	0.915
Academic buoyancy (AB)	4	0.625	0.869	0.869	0.867
Teacher–class communication (TCC)	4	0.712	0.908	0.908	0.907
Teacher–individual communication (TIC)	6	0.685	0.929	0.929	0.929
Peer communication (PC)	5	0.617	0.890	0.890	0.889
Group communication (GC)	5	0.677	0.913	0.913	0.912

#### 3.2.4 Interview outline

To gain deeper insights into students' willingness to communicate and its underlying influences, a semi-structured interview protocol was designed as a supplementary research instrument. The interview questions focused on learners' perceptions and experiences of classroom-based foreign language anxiety, academic buoyancy, and communication behaviors. Eight students were randomly selected for individual, face-to-face interviews, each lasting between 20 and 30 min.

### 3.3 Data analysis

Data were analyzed using SPSS 26.0, AMOS 26.0 and R 4.4.2. Reverse-coded items were first recoded prior to analysis. Reliability testing, descriptive statistics, and Pearson correlation analyses were conducted using SPSS. AMOS was employed for confirmatory factor analysis (CFA), structural equation model (SEM), and testing of the mediation model. McDonald's ω was computed in R using the MBESS package (Hayes and Coutts, 2020). Indirect effects were examined via bias-corrected and accelerated (BCa) bootstrapping with 5,000 resamples.

## 4 Quantitative results

### 4.1 Descriptive statistics and correlational analysis

The descriptive statistics presented in Table 2 show that the mean scores of the four dimensions of foreign language anxiety, academic buoyancy, and the four dimensions of willingness to communicate in the classroom all fell within the range of 2.5 to 3.4, indicating a moderate level.

**Table 2:** Descriptive statistics and inter-construct correlations (√AVE on the diagonal, *N* = 627).

**Variables**	**CA**	**FNE**	**TA**	**GCA**	**AB**	**TCC**	**TIC**	**PC**	**GC**
CA	0.818								
FNE	0.589^**^	0.797							
TA	0.571^**^	0.406^**^	0.809						
GCA	0.535^**^	0.688^**^	0.377^**^	0.758					
AB	−0.395^**^	−0.379^**^	−0.300^**^	−0.346^**^	0.790				
TCC	−0.383^**^	−0.321^**^	−0.373^**^	−0.384^**^	0.470^**^	0.844			
TIC	−0.403^**^	−0.350^**^	−0.362^**^	−0.369^**^	0.488^**^	0.521^**^	0.828		
PC	−0.439^**^	−0.323^**^	−0.327^**^	−0.330^**^	0.491^**^	0.560^**^	0.592^**^	0.785	
GC	−0.433^**^	−0.383^**^	−0.385^**^	−0.360^**^	0.579^**^	0.511^**^	0.554^**^	0.591^**^	0.823
Mean	2.829	2.961	2.794	2.68	3.249	3.315	3.177	3.258	3.24
Standard deviation	0.993	0.861	0.871	0.823	0.775	0.911	0.867	0.866	0.827

CA, Communication Apprehension; FNE, Fear of Negative Evaluation; TA, Test Anxiety; GCA, General Classroom Anxiety; AB, Academic Buoyancy; TCC, Teacher–Class Communication; TIC, Teacher–Individual Communication; PC, Peer Communication; GC, Group Communication.

^**^ *p* < 0.01.

The Pearson correlation results revealed significant correlations among all major variables. Specifically, foreign language anxiety was negatively correlated with academic buoyancy (*r* = −0.395 to −0.300, *p* < 0.01), and also negatively correlated with willingness to communicate (r = −0.439 to −0.321, *p* < 0.01). In contrast, academic buoyancy was positively correlated with willingness to communicate in the classroom (*r* = 0.470–0.579, *p* < 0.01). These findings suggest that lower levels of foreign language anxiety are associated with higher levels of academic buoyancy and a stronger personal willingness to engage in classroom communication in English.

The discriminant validity of the constructs was further confirmed using the Fornell-Larcker criterion (Fornell and Larcker, 1981). As shown in Table 2, the square roots of the AVEs, displayed along the diagonal, were consistently higher than the corresponding inter-construct correlations, demonstrating that all latent dimensions were empirically distinct from one another.

### 4.2 Structural equation modeling: effects of foreign language anxiety and academic buoyancy on willingness to communicate

To examine the structural relationship between foreign language anxiety, academic buoyancy, and willingness to communicate (WTC) in the classroom, a two-step modeling procedure was employed (Anderson and Gerbing, 1988). First, the direct effect of foreign language anxiety on WTC was tested. If the direct effect proved significant, academic buoyancy would be introduced as a potential mediating variable in the subsequent model.

**Figure 1:**
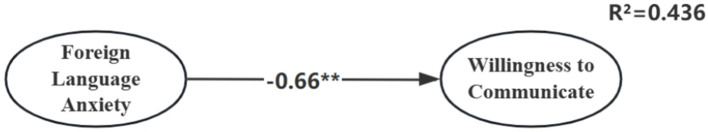
Direct-effect model of foreign language anxiety on willingness to communicate. Coefficients are standardized (**p < 0.01).

The results from the initial model assessing the direct effect showed a good model fit: χ^2^/df = 2.319 (within the acceptable range of 1–3), RMSEA = 0.046 (< 0.05), TLI = 0.930(>0.90), CFI = 0.933 (>0.90), and SRMR = 0.054 (< 0.08) (Hu and Bentler, 1999). The path from foreign language anxiety to WTC was statistically significant (β = −0.660, *p* < 0.001), with the model explaining 43.6% of the variance in WTC (see Figure 1). These results met the prerequisites for further testing of a mediation model.

**Figure 2:**
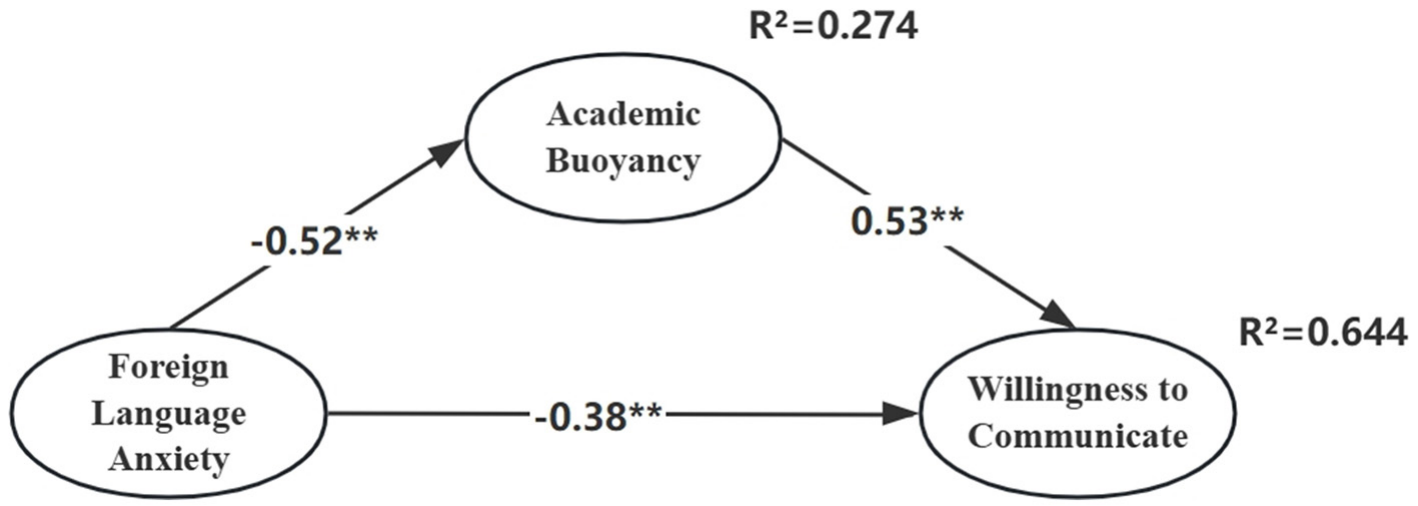
Mediation model with academic buoyancy as a mediator. Coefficients are standardized (**p < 0.01). Foreign language anxiety and willingness to communicate were modeled as second-order constructs (four first-order dimensions each); AB is first-order.

**Note**. Coefficients are standardized (^**^p < 0.01). Foreign language anxiety and willingness to communicate were modeled as second-order constructs (four first-order dimensions each); AB is first-order.

Subsequently, academic buoyancy was introduced as a mediating variable, and a three-variable structural equation model was constructed. Model fit indices indicated a satisfactory model (Hu and Bentler, 1999): χ^2^/df = 2.311, CFI = 0.928, TLI = 0.925, RMSEA = 0.046, and SRMR = 0.053. These results suggested a good overall model fit (see Figure 2). The squared multiple correlation for WTC (R^2^) was 0.644, indicating that 64.4% of the variance was explained by the model. Compared with the direct effect model, the inclusion of academic buoyancy increased the explanatory power by 20.8%.

To determine which structural model best represented the data, three competing specifications were evaluated: a direct-effect model, a full-mediation model, and a partial-mediation model (see Table 3). All three models demonstrated acceptable fit (χ^2^/df = 2.31–2.36, RMSEA < 0.05, SRMR < 0.08), indicating satisfactory model adequacy. Minor differences were found among the models, suggesting comparable levels of overall fit.

**Table 3:** Model Fit comparison among direct, full-mediation, and partial-mediation models.

**Model**	**χ^2^/df**	**CFI**	**TLI**	**RMSEA**	**SRMR**	**R^2^(WTC)**
Direct model	2.319	0.933	0.93	0.046	0.054	0.436
Full mediation model	2.356	0.925	0.922	0.047	0.075	0.576
Partial mediation model	2.311	0.928	0.925	0.046	0.053	0.644

Whether the direct path from foreign language anxiety to willingness to communicate should be retained was evaluated via a nested comparison of mediation models. The partial-mediation model demonstrated superior fit to the full-mediation model (Δχ^2^(1) = 72.027, *p* < 0.001) and was therefore retained. It also accounted for more variance in WTC (R^2^ = 0.644) than the direct-only (R^2^ = 0.436) and full-mediation (R^2^ = 0.584) models. The indirect effect through academic buoyancy was significant (**β**_indirect = −0.279, 95% BCa CI [−0.340, −0.223]). Overall, the partial-mediation model provides the most theoretically and empirically adequate representation of the data; the direct-only model is presented as a baseline.

Based on the partial-mediation model (Figure 2), bias-corrected bootstrapping with 5,000 resamples was performed using AMOS to assess the significance of the direct, indirect, and total effects of each path in the mediation model. The results are as follows: (1) The overall effect of foreign language anxiety on willingness to communicate was −0.662, indicating that for each unit increase in anxiety, students' WTC decreased by 0.662 units, considering both direct and indirect influences. (2) The direct effect was −0.383, and the indirect effect through academic buoyancy was −0.279. This suggests that a one-unit increase in anxiety would originally lead to a 0.383 unit decrease in WTC. However, with academic buoyancy functioning as a positive mediator, the final decrease was mitigated to 0.279 units, indicating that academic buoyancy partially offset the negative influence of anxiety on students' willingness to communicate. (3) The 95% bias-corrected confidence interval for the indirect effect was (−0.340, −0.223), which does not include zero, indicating that the mediating effect of academic buoyancy was statistically significant.

**Figure 3:**
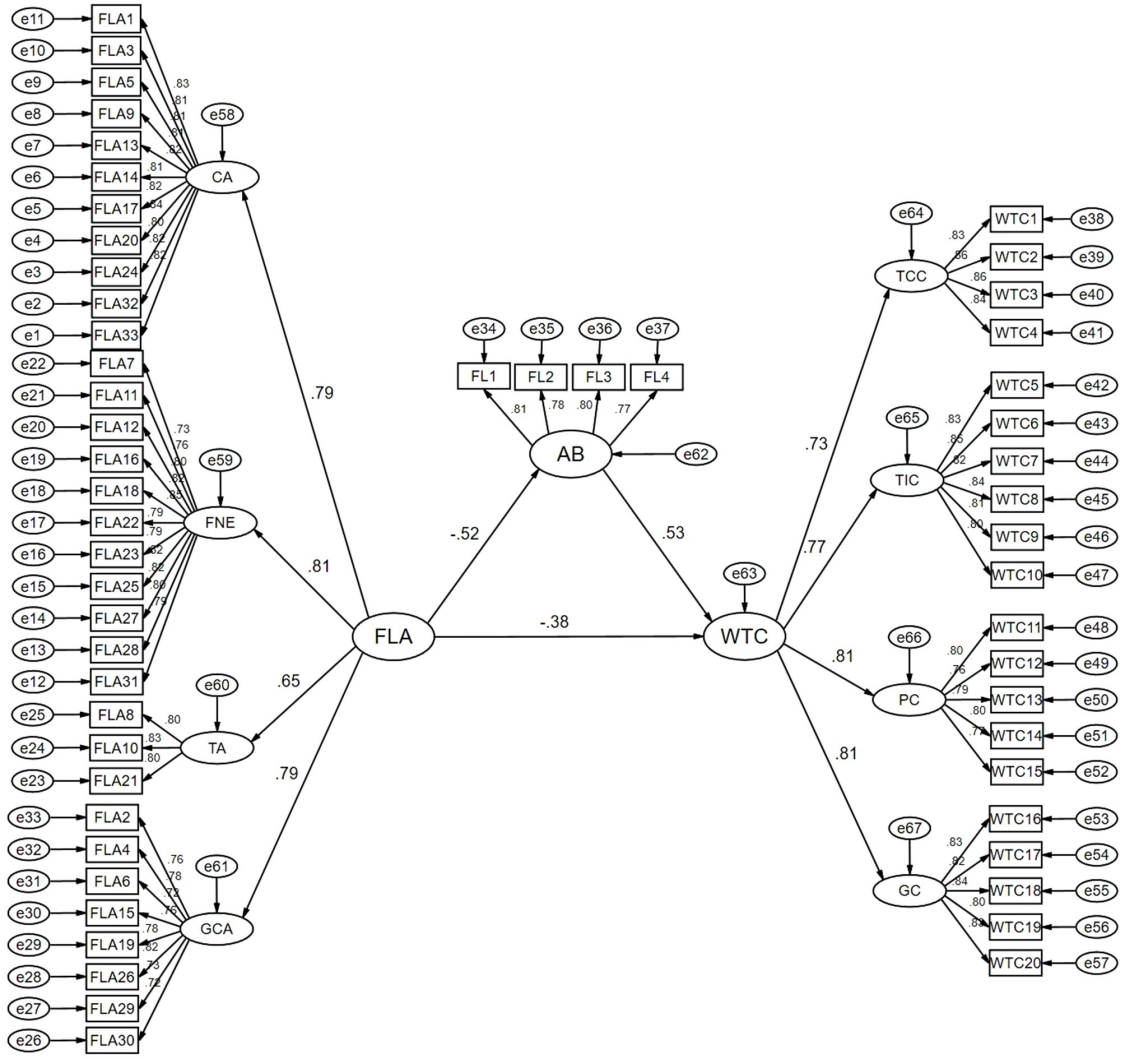
Final partial-mediation model with second-order constructs and standardized loadings. CA, Communication Apprehension; FNE, Fear of Negative Evaluation; TA, Test Anxiety; GCA, General Classroom Anxiety; AB, Academic Buoyancy; TCC, Teacher-Class Communication; TIC, Teacher-Individual Communication; PC, Peer Communication; GC, Group Communication.

To provide a comprehensive view of the final validated model, Figure 3 displays the partial mediation model with second-order constructs and standardized loadings for all observed indicators.

## 5 Qualitative findings

### 5.1 The impact of foreign language anxiety on willingness to communicate

#### 5.1.1 The prevalence and sources of foreign language anxiety

In the interviews, all participants reported experiencing moments in English class where they fully understood the content but found themselves unable to speak. This pattern highlights the widespread presence of foreign language anxiety (FLA) and its suppressive effect on students' willingness to communicate (WTC) in oral tasks. Many students described reactions such as intense nervousness, mental blocks, or sudden blankness during speaking activities. These symptoms were commonly linked to fear of making mistakes, lack of confidence in pronunciation or intonation, and concerns about peer evaluation.

For example, both Student 1 and Student 5 reported feeling highly anxious even when well-prepared. Student 1 recalled, “I was ready, but as soon as the teacher called on me, my mind went blank and my heart was racing.” Similarly, Student 5 shared, “I usually practice a lot, but when it's my turn to speak in class, my heart pounds and I forget the words.” Student 8 added, “I know I can say it, but I just can't open my mouth.” These cases suggest that the source of anxiety lies not in linguistic competence itself, but in emotional interference at the point of expression.

Beyond the fear of errors, some students pointed to more socially constructed sources of anxiety. As Student 1 put it, “I'm afraid that if I make a mistake or my pronunciation is off, people will laugh at me.” Student 4 expressed a similar concern: “Sometimes it's not about saying it wrong, it's about being heard and then labeled as ‘bad at speaking'.” These responses indicate that, under the dual pressure of exam-oriented teaching and peer evaluation, students may come to view speaking in class not as a learning opportunity, but as a social risk.

#### 5.1.2 Strategies for coping with foreign language anxiety

Faced with anxiety, students adopted diverse coping strategies. While some opted for avoidance to reduce emotional pressure, others actively sought ways to manage their anxiety and continue participating. For instance, Student 2 stated, “If I gave a wrong answer last class, I probably wouldn't raise my hand the next time, unless the teacher called on me.” Student 6 exhibited similar avoidance tendencies. In contrast, Student 3 described a more proactive approach: “Even though I feel nervous, if I've prepared well, sometimes I push myself to speak, and then I realize it's not that scary.” Student 7 used rehearsal as a way to lower anxiety: “I memorize a template in advance and mentally recite the first few lines. Once I get the first sentence out, the rest flows more easily.”

These accounts suggest that FLA is a common experience among Chinese high school students in English classrooms. Most participants demonstrated a heightened sensitivity to the potential consequences of making mistakes, which, at least to some extent, undermines their willingness to communicate. However, students' responses varied: while some withdrew from speaking opportunities, others actively regulated their emotional state and prepared compensatory strategies, highlighting a potential space for psychological resilience and adaptive coping in the language learning process.

### 5.2 The mediating role of academic buoyancy

#### 5.2.1 Regulating emotional pressure through academic buoyancy

While foreign language anxiety was commonly observed, some students demonstrated the ability to gradually develop academic buoyancy through repeated engagement with speaking challenges. This buoyancy helped reduce the emotional pressure associated with oral expression. Interview data suggest that academic buoyancy positively influenced students' willingness to communicate through two key pathways: emotional regulation and behavioral strategies.

Some students, through repeated exposure to speaking failures, began to reframe mistakes as a natural part of the learning process, thereby weakening the emotional threat posed by errors. Student 3 reflected, “At first, I felt ashamed when I said something wrong, but then I realized not everyone speaks perfectly, and the teacher doesn't correct me in front of everyone, eventually I stopped being afraid.” Similarly, Student 7 shared, “I used to get corrected a lot, but then I thought, others make mistakes too. After a few times, I got used to it and didn't care as much.” These accounts show that when errors are no longer perceived as threatening, students are more willing to re-engage in classroom communication.

In addition, students developed personal strategies to stabilize their emotional state and reduce anxiety. Student 4 noted, “When I don't speak smoothly, I first scold myself silently, but after a few classes, I forget about it.” Student 8 employed subtle physical techniques to calm themselves before speaking: “I take deep breaths before speaking, and sometimes I tap my fingers under the desk to remind myself not to panic.” Though seemingly minor, these self-regulatory behaviors reflect a shift from passive endurance to active emotional management.

Overall, academic buoyancy enabled students to desensitize the emotional impact of communicative failure and build psychological tolerance toward language setbacks. This capacity stemmed less from linguistic proficiency and more from their ability to emotionally recover and adapt in the face of communicative stress.

#### 5.2.2 Activating willingness to communicate through buoyancy

In addition to emotional regulation, some students used strategic preparation to enhance their sense of control in speaking tasks, thereby increasing their willingness to communicate. As Student 6 explained, “I wrote two possible answers for each question I thought the teacher might ask. If one didn't work, I had a backup. That made me feel more secure.” These forms of pre-planned responses reflect not only a cautious approach to potential failure but also an outward expression of academic buoyancy.

Moreover, positive teacher feedback was identified by several students as a motivating factor for sustained participation. Student 2 recalled, “Sometimes I don't answer well, but if the teacher says, ‘At least you had the courage to speak,' I feel less nervous.” Student 5 added, “When the teacher lets me finish and then says, ‘Actually, what you said was pretty clear,' I feel more confident about speaking next time.” This kind of feedback does not directly correct language errors but instead affirms the communicative attempt itself, helping students reframe classroom speaking as a meaningful and low-risk endeavor.

In this way, academic buoyancy not only enabled students to tolerate emotional pressure but also encouraged them to take initiative, plan ahead, and draw strength from supportive feedback. This internally driven process of regulation and activation serves as a crucial psychological bridge between foreign language anxiety and willingness to communicate.

### 5.3 Additional factors influencing willingness to communicate

In addition to the emotional and personality-based pathway composed of anxiety and academic buoyancy, several students pointed to contextual factors within the classroom that also influenced their willingness to communicate. These included teacher behavior, peer dynamics, and the design of speaking tasks.

#### 5.3.1 Teacher Style, Peer Reactions, and Classroom Atmosphere

Some participants reported that strict teacher styles or peer-driven evaluative climates led to a marked decrease in their willingness to speak. Student 1 commented plainly, “When the teacher is too strict, I just don't dare to speak.” Student 6 observed, “In our class, very few people speak English. It feels awkward if you're the only one talking.” In contrast, a relaxed classroom environment with high error tolerance was associated with greater willingness to participate. As Student 4 explained, “Our class atmosphere is good. Even if you make a mistake, it's not a big deal, so I feel more willing to speak.”

These accounts suggest that a sense of psychological safety, cultivated through teacher attitude and peer acceptance, is a foundational condition for encouraging student participation. In traditional Chinese classrooms where instruction often prioritizes written accuracy and exam performance, limited interaction and rigid evaluative standards may heighten students' caution, thereby reducing their communicative engagement.

#### 5.3.2 The role of speaking task design

The structure of speaking tasks also shaped students' perceptions of pressure and their willingness to communicate. Several students expressed a clear preference for speaking tasks with greater flexibility and lower performance demands. Student 6 noted, “I'm more willing to speak in small group discussions.” Student 7 added, “Not every task has to require full English sentences. It feels more relaxed when making mistakes is okay.”

These responses indicate that open-ended, low-stakes speaking tasks help lower the psychological burden of language output. When teachers reduce linguistic barriers or offer supportive scaffolding in task design, students may experience less anxiety and show increased willingness to communicate.

## 6 Discussion

### 6.1 The direct effect of foreign language anxiety on willingness to communicate in efl classroom

The results of this study demonstrate that foreign language anxiety (FLA) significantly and negatively predicts students' willingness to communicate (WTC) in the EFL classroom. As (MacIntyre et al. 1998) argued, WTC in a second language is context-dependent. While many previous studies examined general WTC without specifying the communicative setting, the present study focused specifically on the classroom context. The findings are consistent with earlier research showing a negative association between anxiety and WTC (Kalsoom et al., 2020; Liu, 2018), thereby extending the empirical support for this relationship and underscoring the classroom as a representative and frequent site of second language use among students.

Beyond the significant quantitative association between FLA and WTC, the interview data provided further insight into how anxiety disrupted students' classroom communication. Several participants reported that, despite fully understanding the content and being well-prepared, they often struggled to speak due to nervousness, confusion, or mental blankness. These accounts suggest that anxiety may inhibit verbal output by functioning as emotional interference, with its impact stemming not from a lack of linguistic competence but from the psychological burden embedded in the act of expression. This qualitative evidence contributes to a deeper understanding of the mechanisms through which anxiety undermines students' communicative willingness.

These findings can also be interpreted within the framework of the WTC pyramid model, which posits that second language communication is shaped by both individual and situational factors (MacIntyre et al., 1998). L2 communication occurs in specific contexts and is sensitive to the social environment. In this regard, classroom-based foreign language anxiety, as a prevalent academic emotion, can permeate students' learning experiences and influence their perceptions of the learning environment. Under the influence of anxiety, students are more likely to restrict their verbal expression (MacIntyre and Gardner, 1991), experience a sense of reduced support and psychological insecurity, and consequently exhibit lower WTC.

This result offers practical implications for classroom language instruction. Teachers should pay close attention to students' emotional states during lessons, offer positive emotional feedback, and foster a psychologically supportive learning atmosphere. Such practices can enhance students' sense of safety in second language interactions and encourage them to use the language more actively, thereby promoting the development of their communicative competence in classroom settings.

### 6.2 The mediating role of academic buoyancy

The results indicated that academic buoyancy partially mediated the relationship between foreign language anxiety and students' willingness to communicate (WTC) in the EFL classroom. Regarding the antecedent variable, foreign language anxiety was found to significantly and negatively predict academic buoyancy (β = −0.524, *p* < 0.01), which is consistent with the “5Cs” amodel proposed by (Martin et al. 2010) and supports prior findings on the negative correlation between anxiety and academic buoyancy (Zheng et al., 2023). This study also contributes by further exploring their potential causal relationship. Interestingly, in contrast to most studies, (Liu et al. 2025) reported that anxiety can positively predict academic buoyancy under certain conditions. This divergence may be attributed to the type of anxiety examined, facilitating anxiety rather than debilitating anxiety (Scovel, 1978). While anxiety is typically perceived as detrimental, moderate levels of anxiety may in some cases activate learners' adaptive capacities, which in turn enhance their academic buoyancy and support coping with academic stress.

In terms of the outcome variable, academic buoyancy positively predicted students' willingness to communicate in the classroom (β = 0.532, *p* < 0.01), confirming the role of positive psychological traits in facilitating L2 communication. This finding aligns with and extends prior research on resilience and WTC. For instance, (Khajavy et al. 2019) found that resilience, as part of psychological capital, was positively associated with WTC among Iranian learners. (Trigueros et al. 2024) further confirmed that academic resilience significantly predicted WTC in a secondary education context, and (Abdullaeva et al. 2024) provided experimental evidence that resilience can be enhanced through technological literacy interventions to boost WTC in AI-based assessments. Together, these studies underscore the importance of psychological adaptability in shaping learners' communicative engagement. Building on this foundation, the present study focuses on academic buoyancy, a concept more specific to students' daily academic setbacks. Compared to resilience, which typically concerns major adversity, buoyancy may offer a more precise lens to understand and support learners' communication willingness in regular classroom settings, especially within the framework of positive psychology.

The practical significance of these findings lies in the recognition that academic buoyancy not only helps students cope with academic pressure and everyday setbacks, such as exam failure, but also enhances their emotional regulation and self-confidence in the classroom (Martin and Marsh, 2008; Putwain et al., 2012). Students with high levels of buoyancy are more likely to recover quickly from setbacks, maintain a positive outlook, and actively engage in classroom interaction, leading to more frequent communication with both teachers and peers. In addition, it promotes the development of their language expression skills, strengthens their social willingness, and fosters a stronger sense of classroom belonging. Academic buoyancy also yields emotional benefits by reducing anxiety, fostering composure, and facilitating positive classroom experiences. In this respect, buoyant learners are not only academically persistent but also emotionally resilient, able to regulate negative affect and maintain constructive engagement in communicative tasks (Martin and Marsh, 2008; Putwain et al., 2023; Yun et al., 2018). This view is consistent with Seligman's (2011a,b) argument that resilience, as a trainable capacity, enables individuals to recover from adversity and develop adaptive coping strategies, suggesting that academic buoyancy can likewise be cultivated through classroom practices. These emotional benefits highlight the adaptive role of buoyancy in mitigating the affective barriers to classroom communication.

A key outcome is that fostering students' academic buoyancy not only contributes to their academic performance but also improves their WTC and participation in classroom settings. As (MacIntyre et al. 1998) noted, learners' willingness to communicate has a direct impact on actual communicative behavior and plays a vital role in L2 acquisition outcomes. In this regard, the positive predictive effect of academic buoyancy on WTC empirically highlights its capacity to regulate emotions, support psychological recovery, and enhance learners' everyday adaptability, ultimately providing valuable psychological resources that help students better cope with communicative challenges in the second language learning process.

These findings affirm the theoretical relevance of positive psychological capital theory and emotional regulation theory in the context of second language acquisition. As a stable positive psychological construct, academic buoyancy offers both cognitive and emotional support when learners face academic stress or anxiety (Yun et al., 2018), thereby mitigating the disruptive effects of emotional states on classroom communicative behavior. Learners with higher levels of buoyancy tend to possess stronger emotional regulation abilities (Martin and Marsh, 2008), enabling them to manage anxiety more effectively, maintain positive classroom engagement, and enhance their willingness to interact with others.

This pattern is also consistent with Krashen's (1985) Affective Filter Hypothesis, which posits that emotional states influence both language input and output. The emotional stability conferred by academic buoyancy may help reduce the filtering effects of anxiety, enhance language processing efficiency, and improve classroom interaction. This mechanism aligns with the view proposed by (Abbas et al. 2024), who emphasized emotion-regulating role of positive psychological traits in managing negative affective states. Beyond Krashen's (1985) affective filter framework, the present findings also resonate with the PERMA model of wellbeing (Seligman, 2011a,b). Within this framework, resilience is regarded as a foundational capacity for sustaining wellbeing. Academic buoyancy, as a domain-specific form of resilience, appears to play this role in language learning by mitigating the negative impact of foreign language anxiety and sustaining learners' communicative engagement. Specifically, the mediating role of buoyancy observed in this study reflects its function in maintaining emotional stability (positive emotion), encouraging sustained participation in classroom discourse (engagement), and supporting incremental communicative accomplishments (accomplishment). These links illustrate how buoyancy, beyond reducing anxiety, contributes to students' willingness to communicate through mechanisms consistent with broader wellbeing processes (Oxford, 2016a,b; Wong et al., 2022).

Qualitative evidence also revealed the mechanisms through which academic buoyancy positively influences students' willingness to communicate. Several interviewees reported that, after repeated setbacks in oral expression, they gradually came to view making mistakes as non-threatening. By employing self-regulatory strategies, such as deep breathing or preparing alternative responses in advance, they were able to reduce anxiety and enhance their sense of control. This shift from emotional recovery to proactive engagement illustrates how academic buoyancy supports willingness to communicate through both emotional regulation and behavioral activation, offering contextual evidence that complements the mediating pathway identified in the quantitative analysis.

Collectively, the findings reveal that foreign language anxiety influences classroom willingness to communicate through both direct and indirect pathways, with academic buoyancy serving as a partial mediator. These results suggest that both foreign language anxiety (as an emotional variable) and academic buoyancy (as a positive psychological resource) independently predict learners' communicative willingness. The study thus extends the WTC pyramid model by elucidating how personality traits and emotional factors interact to shape communication behavior, addressing a gap in prior research regarding the joint effects of these two variable types (Wang, 2023). In line with the recent turn toward wellbeing in applied linguistics, the positive influence of academic buoyancy on WTC underscores its contribution to learners' overall psychological health and flourishing (Abbas et al., 2024; Oxford, 2016a,b; Wong et al., 2022). By supporting wellbeing in everyday classroom contexts, buoyancy provides learners with sustainable psychological resources that strengthen both language development and personal growth. Beyond this positive psychology perspective, existential views emphasize the role of meaning-making in coping with adversity. Such a view resonates with Frankl's (1985) proposition that meaning emerges not from the absence of adversity, but from one's attitude toward it. Within the context of language learning, anxiety and setbacks can be reframed as opportunities for psychological growth. When supported by internal resources such as academic buoyancy, emotional challenges may not hinder communication but instead foster resilience, agency, and deeper engagement in classroom interaction.

### 6.3 Other factors influencing willingness to communicate

Several interviewees reported that teacher style, peer response, task design, and the overall classroom atmosphere significantly influenced their willingness to communicate (WTC). This finding aligns with the conclusions of (Kun et al. 2020) and (Zerey and Cephe 2020), who identified “teacher support” “student cohesiveness” and “task orientation” as key dimensions of the classroom environment and found these factors to be positively associated with students' WTC. Consistent with the qualitative findings of the present study, these three elements were also frequently mentioned by participants as influential. Some students indicated that positive and individualized feedback from teachers enhanced their confidence in speaking, whereas strict teacher attitudes or judgmental peer behavior often led them to remain silent. These observations suggest that critical social elements of the classroom environment can indirectly shape students' communicative behaviors by influencing their emotional experiences and self-perceptions. Accordingly, teachers should recognize the contextual role of their everyday interactions and support strategies in shaping learners' WTC and strive to foster a supportive classroom climate.

In addition, the present findings partly converge with those of (Joe et al. 2017), though key differences exist. While their study focused primarily on the relational dimension of classroom dynamics, proposing that a socially supportive climate (characterized by teacher support and mutual respect) enhances WTC by fulfilling students' basic psychological needs for autonomy, relatedness, and competence, the current study extends this perspective by highlighting the joint predictive power of multiple social-contextual factors within the classroom ecology, including teachers, peers, and tasks. This broader ecological view resonates with Van Lier's (2010) theory of language learning as an ecological process, which posits that language development does not occur in cognitive isolation but is embedded within a dynamic system involving multiple interacting variables such as teachers, peers, tasks, time, and space.

## 7 Conclusion and pedagogical implications

This study adopted a mixed-methods approach, combining a structural equation model and semi-structured interviews with senior high school students, to examine the mechanism through which foreign language anxiety and academic buoyancy influence students' willingness to communicate in the classroom. The results confirmed the mediating role of academic buoyancy in the relationship between anxiety and willingness to communicate, highlighting its function as a mediating mechanism through which positive psychological resources help transform anxiety into manageable experiences.

Moreover, The finding that academic buoyancy positively predicts classroom WTC highlights a personality trait that combines emotional regulation and psychological adaptability into the research framework of WTC. This expands the WTC pyramid model by clarifying the interaction between personality and affective factors. The study thus offers both theoretical support for understanding emotional regulation mechanisms in second language learning and practical guidance for classroom-based psychological interventions. Specifically, teachers can draw on this framework to help students build academic buoyancy, reduce classroom anxiety, cultivate a positive mindset, and develop effective coping strategies. These efforts can ultimately increase students' willingness to communicate in second language contexts. To translate these theoretical insights into pedagogical practice, the following section outlines targeted instructional recommendations for promoting students' WTC.

Drawing on questionnaire data and interview insights, this study proposes targeted pedagogical recommendations across three dimensions, namely teachers, students, and classroom ecology, with the aim of enhancing students' WTC in foreign language classrooms through multidimensional coordination.

At the teacher level, it is essential to reconceptualize the role of errors in classroom interactions, not merely as issues to be corrected, but as opportunities for learning and personal growth. When students make mistakes, teachers are encouraged to prioritize emotional acceptance before providing instructional feedback, thus fostering a sense of psychological safety. Moreover, feedback should shift away from outcome-based judgments toward an emphasis on the student's effort and engagement in the communicative process, which can alleviate pressure and reduce fear of negative evaluation. Crucially, instructional objectives should extend beyond linguistic competence to include the cultivation of emotional resilience and stress regulation skills, thereby supporting students' long-term psychological development.

From the student perspective, pedagogical support should focus on activating learner agency. Students can be guided to develop personalized emotional regulation strategies, such as deep breathing or mindfulness-based preparation, to manage pre-task anxiety. They may also be encouraged to reflect on past experiences where they persisted despite communicative setbacks, helping them derive positive meaning and foster self-affirmation. These reflective processes can serve as key pathways for building academic buoyancy.

At the classroom level, the creation of a psychologically inclusive environment is particularly critical. Teachers may promote a collective classroom culture that views verbal expression as valuable and mistakes as understandable, thereby normalizing communicative risks. Establishing peer support structures, such as fixed or rotating “communication partners,” can further enhance students' sense of safety and belonging. In addition, organizing communicative tasks of increasing difficulty allows students to experience different levels of pressure, enabling them to build confidence and adaptability over time.

While this study offers some insights into the interplay between foreign language anxiety, academic buoyancy, and WTC, several areas remain open for further exploration. First, the sample was limited to high school students from a single region. Future research could include students from other educational stages, such as junior secondary or post-secondary levels, and broader regions to enhance the external validity and generalizability of the results. Second, the study employed a cross-sectional self-report design. Future investigations may benefit from experimental or longitudinal approaches to further explore the dynamic relationships among foreign language anxiety, academic buoyancy, and WTC. Furthermore, the qualitative findings suggest that classroom-related contextual factors, such as classroom climate, peer interactions, and instructional style, may also play a significant role in shaping students' WTC. These insights point to promising directions for future research, including the integration of classroom ecology variables into extended models of WTC, as well as further qualitative investigations to uncover nuanced pathways through which situational factors interact with learner psychology.

## Data Availability

The raw data supporting the conclusions of this article will be made available by the authors, without undue reservation.
