# Supplementary material for: The relations among foreign language anxiety, academic buoyancy and willingness to communicate in EFL classroom
Source: Front Psychol. 2025 Oct 28;16:1634054. doi: 10.3389/fpsyg.2025.1634054 (PMC12602425; doi:10.3389/fpsyg.2025.1634054)
Supplement: Supplementary file 1 [file Data_Sheet_1.pdf]

Supplementary Table A-Standardized Loadings, Composite Reliability, and Convergent Validity of All Measured Constructs

This table presents the standardized factor loadings (λ), average variance extracted (AVE), composite reliability (CR), McDonald's ω, and Cronbach's α for all latent constructs included in the structural model.

Construct	Items		Standardized loadings (λ)	AVE	CR	McDonald's ω	Cronbach's α
Academic Buoyancy（AB)	AB1		0.807				
	AB2		0.783	0.625	0.869	0.869	0.867
	AB3		0.809				
	AB4		0.761				
Foreign Language Anxiety(FLA)	Communication Apprehension（CA)	FLA1	0.825				
		FLA3	0.814				
		FLA5	0.811				
		FLA9	0.813				
		FLA13	0.822				
		FLA14	0.811	0.669	0.957	0.957	0.957
		FLA17	0.816				
		FLA20	0.836				
		FLA24	0.805				
		FLA32	0.821	0.583	0.847		
		FLA33	0.821				
	Fear of Negative Evaluation(FNE)	FLA7	0.73				
		FLA11	0.761				
		FLA12	0.8				
		FLA16	0.814	0.635	0.950	0.951	0.95
		FLA18	0.847				
		FLA22	0.791				
		FLA23	0.79				
		FLA25	0.818				

		FLA27	0.822						
		FLA28	0.796						
		FLA31	0.791						
		FLA8	0.806						
	Test Anxiety(TA)	FLA10	0.817	0.655		0.851		0.850	0.849
		FLA21	0.805						
		FLA2	0.757						
		FLA4	0.776						
		FLA6	0.721						
		FLA15	0.759						
	General Foreign Language Anxiety(GCA）	FLA19	0.775	0.574		0.915		0.915	0.915
		FLA26	0.812						
		FLA29	0.734						
		FLA30	0.724						
Willingness to Communicate(WTC)	Teacher–Class Communication(TCC)	WTC1	0.825	0.712	0.609	0.908	0.862	0.908	0.907

	WTC2	0.858				
	WTC3	0.858				
	WTC4	0.833				
	WTC5	0.835				
	WTC6	0.854				
Teacher–Individual Communication(TIC)	WTC7	0.826	0.685	0.929	0.929	0.929
	WTC8	0.837				
	WTC9	0.81				
	WTC10	0.801				
	WTC11	0.799				
	WTC12	0.765				
Peer Communication(PC)	WTC13	0.786	0.617	0.890	0.890	0.889
	WTC14	0.805				
	WTC15	0.773				
	WTC16	0.831				
	WTC17	0.816				
Group Communication(GC)	WTC18	0.834	0.677	0.913	0.913	0.912
	WTC19	0.799				
	WTC20	0.833				
